# Dose-related effects of acute cycling on executive function in children with autism spectrum disorder: a within-subject repeated-measures study

**DOI:** 10.3389/fpsyg.2026.1902127

**Published:** 2026-07-30

**Authors:** Yifan Shi, Funing Gao, Zhimei Liu, Kelong Cai, Zhiyuan Sun, Yifan Xu, Liye Zou, Aiguo Chen

**Affiliations:** 1 School of Sport and Brain Health, Nanjing Sport Institute, Nanjing, China; 2 College of Physical Education, Yangzhou University, Yangzhou, China; 3 Children’s Hospital of Nanjing Medical University, Nanjing, China; 4 Affiliated Hospital of Yangzhou University, Yangzhou, China; 5 University of Physical Education and Sport, Gdansk, Poland; 6 Body-Brain-Mind Laboratory, School of Psychology, Shenzhen University, Shenzhen, China

**Keywords:** autism spectrum disorder, dose–response relationship, executive function, exercise, inhibition, working memory

## Abstract

**Background:**

Exercise has shown promise for supporting executive function in children with autism spectrum disorder (ASD), but findings remain inconsistent, partly because exercise dose has not been examined systematically. This study examined the immediate effects of acute cycling with different combinations of intensity and duration on executive function in children with ASD.

**Methods:**

A within-subject repeated-measures design was used. Thirty children with ASD completed one non-exercise sedentary control session at enrollment, followed by four counterbalanced cycling sessions arranged in a 2 × 2 exercise-dose matrix: 20 min low-intensity, 40 min low-intensity, 20 min moderate-intensity, and 40 min moderate-intensity cycling. Exercise intensity was monitored using the heart rate reserve method. Selected executive function components were assessed after each condition using the Early Years Toolbox, including the Go/No-Go task for inhibitory control and the Mr. Ant task for working memory. Repeated-measures analyses of variance were conducted, followed by FDR-corrected planned comparisons between each exercise condition and the sedentary control condition.

**Results:**

Significant condition effects were observed for Go reaction time (*p* = 0.024, *partial η^2^* = 0.092), No-Go reaction time (*p* = 0.006, *partial η^2^* = 0.116), Go accuracy (*p* = 0.046, *partial η^2^* = 0.080), No-Go accuracy (*p* = 0.006, *partial η^2^* = 0.115), and the inhibitory-control composite score (*p* < 0.001, *partial η^2^* = 0.405). Moderate-intensity exercise showed the clearest post-session advantages relative to sedentary control, with the 40-min moderate-intensity condition showing the most consistent pattern across inhibitory-control outcomes. For the inhibitory-control composite score, all four exercise conditions were higher than sedentary control. No significant condition effect was found for working memory (*p* = 0.353, *partial η^2^* = 0.037).

**Conclusion:**

Acute cycling may have selective short-term effects on selected executive function components in children with ASD. Moderate-intensity cycling, particularly a 40-min session under the present protocol, was associated with more favorable inhibitory-control performance, whereas immediate changes in working memory were not observed. These findings should be interpreted in light of the non-counterbalanced sedentary control session.

**Clinical trial registration:**

https://www.chictr.org.cn/showproj.html?proj=248491, ChiCTR2400091872.

## 1 Introduction

Autism spectrum disorder (ASD) is an early-onset and lifelong neurodevelopmental condition characterized by persistent difficulties in social communication and social interaction, together with restricted and repetitive patterns of behavior, interests, or activities (American Psychiatric Association, 2022; Lord et al., 2018). In recent decades, the reported prevalence of ASD has increased substantially, making it a major public health concern for children, families, schools, and healthcare systems (Santomauro et al., 2025; Zeidan et al., 2022). Beyond its core diagnostic symptoms, ASD is commonly accompanied by motor difficulties (Fournier et al., 2010), reduced physical activity participation (Li et al., 2021), and cognitive impairments (Demetriou et al., 2018). Among these associated difficulties, executive-function deficits have received increasing attention because they may affect children’s everyday learning, behavioral regulation, and adaptive functioning (Gardiner and Iarocci, 2018; Liang et al., 2022).

Executive function (EF) refers to a set of higher-order cognitive processes that support goal-directed behavior by regulating attention, memory, and behavioral responses (Diamond, 2013). Core EF components typically include inhibitory control, working memory, and cognitive flexibility (Miyake et al., 2000). Inhibitory control allows individuals to suppress dominant or inappropriate responses and resist interference from irrelevant stimuli, whereas working memory supports the temporary storage and manipulation of information during ongoing tasks. These functions are important for classroom learning (Best et al., 2011), self-regulation (Blair and Razza, 2007), social interaction, and daily problem solving (Gardiner and Iarocci, 2018). Children with ASD often experience difficulties in these domains, and such difficulties may be associated with repetitive behaviors, social adaptation, emotional regulation, and behavioral adjustment (Fong and Iarocci, 2020; Gardiner and Iarocci, 2018; Hill, 2004; Iversen and Lewis, 2021). Therefore, identifying feasible and developmentally appropriate approaches to support EF in children with ASD has both theoretical and practical significance.

Physical exercise has increasingly been recognized as a feasible, low-cost, and nonpharmacological approach for promoting cognitive and behavioral functioning in children with ASD (Bremer et al., 2016; Healy et al., 2018). Compared with many clinic-based interventions, exercise can be implemented in school, rehabilitation, and family settings and may be particularly relevant for children who also show motor delays or reduced physical activity participation (Kangarani-Farahani et al., 2024; Liang et al., 2020). Existing studies suggest that both chronic and acute exercise may have positive effects on cognitive performance, including EF (Liang et al., 2022; Liu et al., 2020; Pan et al., 2017). Acute exercise is of particular practical interest because it can be delivered within a single session and may produce short-term changes in cognitive performance that could support subsequent learning, therapy, or behavioral regulation (Chang et al., 2012). The cognitive effects of acute exercise may be partly explained by transient biological and neurophysiological changes. A single bout of aerobic exercise can alter physiological arousal and catecholaminergic activity, which may influence attention allocation, response readiness, and executive control (McMorris, 2016). Acute exercise may also increase cerebral blood flow and oxygen delivery, thereby changing the neural context in which executive tasks are performed (Shirzad et al., 2022). Because executive functions depend strongly on prefrontal and frontoparietal control networks, exercise-induced changes in cerebral perfusion, cortical activation, and cortical excitability may be particularly relevant for tasks requiring response inhibition and working memory (Hillman et al., 2009; Morris et al., 2020). In children with ASD, exploratory evidence has also linked acute exercise to changes in prefrontal oxygenation and inhibitory-control performance, suggesting that neurophysiological responses may contribute to post-exercise cognitive changes in this population (Bremer et al., 2020). More broadly, recent cognitive-motor rehabilitation studies have highlighted the potential role of combined physical and cognitive engagement in improving executive processes and neurophysiological outcomes in clinical populations, although such evidence is not specific to ASD or acute cycling (Deodato et al., 2024).

However, findings regarding the immediate cognitive effects of acute exercise in children with ASD remain inconsistent (Bremer et al., 2020; Ludyga et al., 2024). One possible reason is that exercise dose has not been systematically examined. Exercise intensity and duration are two key dose parameters, yet previous studies often used a single exercise condition or varied several intervention components simultaneously (Chang et al., 2012; Soga et al., 2016; Williams et al., 2019). This is important because the biological and neurophysiological pathways described above are likely to vary according to exercise dose. For example, different intensity levels may produce different degrees of arousal, cardiovascular response, and cortical activation, while different durations may influence the accumulation or dissipation of exercise-induced physiological changes, as well as attention, fatigue, and behavioral regulation during or after exercise (Howie et al., 2015; McMorris, 2016; Sudo et al., 2022; Williams et al., 2019). In the present study, low- and moderate-intensity exercise were selected because these intensities are more feasible and safer for repeated acute testing in young children with ASD than vigorous exercise. Low-intensity exercise may be more tolerable for younger children or those with limited exercise experience, whereas moderate-intensity exercise has been commonly used in pediatric and ASD acute-exercise studies and may provide a stronger but still manageable physiological stimulus (Bremer et al., 2020; Ludyga et al., 2024; Soga et al., 2016). The 20-min duration was selected because brief bouts of approximately this length have been used in prior acute-exercise studies involving children, including children with ASD, and may be more feasible in clinical, school, or therapeutic contexts (Bremer et al., 2020; Howie et al., 2015; Ludyga et al., 2024). The 40-min duration was included as a longer but still time-limited condition to examine whether a more sustained aerobic stimulus would produce more stable post-session executive-function effects, while remaining within a supervised and clinically monitored protocol. These possibilities suggest that the cognitive effects of acute exercise should be examined across different dose combinations rather than inferred from a single exercise prescription (Chang et al., 2012; Liang et al., 2022). In addition, because inhibitory control and working memory involve related but distinguishable cognitive processes, examining both outcomes may help determine whether acute exercise effects are generalized across EF measures or more evident in specific aspects of EF.

Cycling was selected in the present study primarily because it permits relatively precise control of exercise dose, especially intensity and duration. In stationary/ergometer cycling, workload can be adjusted according to heart-rate responses, and the movement pattern can be kept relatively consistent across sessions, making it suitable for isolating acute dose-related effects (Dkaidek et al., 2023; Martins et al., 2021; Paradiso et al., 2013). However, this choice should be understood within the broader ASD exercise literature. Recent evidence suggests that cognitively engaging, skill-based, or game-like activities, such as mini-basketball, augmented-reality motor games, and cycling activities involving spatial updating or dynamic balance, may produce broader executive-function benefits than simpler stationary cycling protocols (Hou et al., 2024; Nekar et al., 2022; Tse et al., 2026; Wang et al., 2020). Therefore, the present protocol was designed to maximize experimental control over exercise dose rather than to test the most cognitively enriched exercise modality. This trade-off should be considered when interpreting the size and generalizability of the observed effects.

Accordingly, the present study examined the immediate effects of acute cycling with different combinations of intensity and duration on EF in children with ASD. Children first completed a non-exercise sedentary control session and then completed four counterbalanced cycling sessions arranged in a 2 × 2 dose matrix: 20 min low-intensity, 40 min low-intensity, 20 min moderate-intensity, and 40 min moderate-intensity cycling. Inhibitory control and working memory were assessed using the Go/No-Go and Mr. Ant tasks from the Early Years Toolbox. The aim was to determine whether acute exercise produces dose-related changes in EF in children with ASD and to identify a potentially suitable dose for short-term cognitive facilitation.

## 2 Methods

### 2.1 Participants

Children with ASD were recruited from the Children’s Hospital Affiliated with Nanjing Medical University. An *a priori* sample-size calculation was performed in G*Power 3.1.9 using repeated-measures analysis of variance (ANOVA) for within-subject factors (Faul et al., 2009). Parameters were set at *α* = 0.05, power = 0.80, and effect size = 0.25, which indicated a minimum required sample of 21 participants. The effect size was based on the previous study on the impact of exercise intervention on the executive function of individuals with autism (Liang et al., 2022). To account for potential attrition and invalid data, 30 children with ASD were screened and enrolled, and all 30 were retained for the final analyses.

The inclusion criteria were as follows: (1) a confirmed diagnosis of ASD according to the Diagnostic and Statistical Manual of Mental Disorders, Fifth Edition (DSM-5); (2) age between 3 and 12 years; (3) developmental quotient or intelligence quotient ≥70; (4) ability to follow simple instructions; and (5) written informed consent from a parent or legal guardian.

The exclusion criteria were as follows: (1) a clear history of head trauma; (2) neurological disorders or psychiatric illness other than ASD; (3) hearing or visual impairment; (4) recent use of medication affecting the central nervous system; (5) physical disability or severe sensory/motor impairment that would interfere with physical activity; and (6) recent participation in exercise-related rehabilitation or intervention programs.

ASD symptom severity was characterized using the Childhood Autism Rating Scale (CARS). Medical records and parent reports were reviewed to identify medication use and co-occurring clinical diagnoses. Children with recent use of medication affecting the central nervous system or with neurological or psychiatric disorders other than ASD, including clinically diagnosed ADHD, were excluded.

### 2.2 Experimental design and procedure

This study used a within-subject repeated-measures design consisting of one non-exercise sedentary control session and four acute cycling sessions arranged in a 2 (intensity: low, moderate) × 2 (duration: 20 min, 40 min) dose matrix (Liu et al., 2020; Van Den Berg et al., 2018). All participants first completed the sedentary control session at enrollment. During this session, children remained seated in the same intervention room used for the exercise sessions and were allowed to view age-appropriate picture books to maintain a quiet resting state.

The duration of the sedentary control session was not fixed but was determined by heart-rate stabilization. Heart rate was continuously monitored, and executive-function testing began after the child’s heart rate had stabilized, and no obvious behavioral agitation was observed. Because the sedentary control session was designed to standardize the physiological resting state rather than to match the duration of the exercise sessions, the exact time required to reach heart-rate stabilization was not systematically recorded.

After the sedentary control session, each participant completed four cycling sessions on separate days: 20 min of low-intensity cycling, 40 min of low-intensity cycling, 20 min of moderate-intensity cycling, and 40 min of moderate-intensity cycling. The order of the four exercise sessions was counterbalanced using a Latin square design to minimize practice and order effects across exercise doses (Bradley, 1958; Lewis, 1989; Richardson, 2018). Each session was completed at the same time of day, with a minimum interval of 7 days between sessions to reduce the influence of circadian rhythm, accumulated fatigue, and carryover effects from the previous session (Hahn et al., 2012; Jones and Kenward, 2014; Munnilari et al., 2024).

Before participation, all children were diagnosed by qualified physicians. The investigator explained the study purpose and procedures to the guardians and obtained written informed consent. Demographic information and potential confounders were collected before testing. Executive-function assessments were performed after the sedentary control session and after each exercise session.

The study was approved by the Human Research Ethics Committee of Nanjing Sport Institute (RT-2024-10). The broader doctoral research project, including the present Study 1, was prospectively registered in the Chinese Clinical Trial Registry on 5 November 2024 (ChiCTR2400091872), before the enrollment of the first participant on 1 March 2025. The public registration record covers a broader project examining short-term exercise interventions in children with ASD, including an individual aerobic cycling intervention, and includes executive function as one of the registered outcome domains. The present manuscript reports the acute cycling dose component and selected executive-function outcomes from Study 1 of this registered project.

### 2.3 Behavioral assessments

#### 2.3.1 Assessment of ASD symptoms

All participants had been clinically diagnosed with ASD before enrollment. Initial screening followed DSM-5 criteria (American Psychiatric Association, 2022). The Childhood Autism Rating Scale (CARS) was used to characterize ASD symptom severity (Schopler et al., 1980). The CARS contains 15 items scored on a 1–4 scale, yielding a total score from 15 to 60, with higher scores indicating greater symptom severity.

All clinical assessments were completed by licensed physicians from the Children’s Hospital Affiliated with Nanjing Medical University.

#### 2.3.2 Assessment of executive function

Executive function was assessed using the Early Years Toolbox cognitive battery (Howard and Melhuish, 2017). The Mr. Ant task was used to assess working memory, and the Go/No-Go task was used to assess inhibitory control. The Card Sorting task, which is typically used to assess cognitive flexibility, was not administered because children with ASD in this age range were expected to have difficulty understanding the instructions (Luo et al., 2023; Zhou et al., 2025). Therefore, EF was operationalized in the present study as inhibitory control and working memory rather than as the full set of core EF components. After each assessment, data were automatically uploaded to the software database.

**Figure 1:**
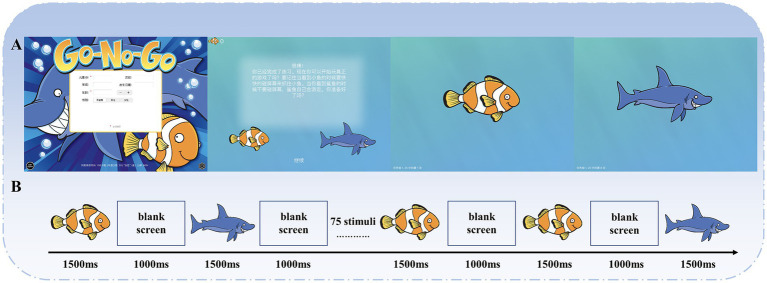
Example of the inhibitory control task paradigm. **A** shows the paradigm instructions and examples of picture stimuli; **B** shows the flowchart of the formal test.

In the Go/No-Go task, fish and sharks appeared on the screen. Participants were instructed to tap the screen when they saw a fish (Go trial; 80% of trials) and to withhold their response when they saw a shark (No-Go trial; 20% of trials). Before the formal test, participants completed 5 Go practice trials, 5 No-Go practice trials, and 10 mixed practice trials. The formal task comprised 75 stimuli presented in pseudorandom order. Each stimulus remained on the screen for 1,500 ms, and the interstimulus interval was 1,000 ms (see Figure 1).

Inhibitory control was evaluated using Go reaction time, No-Go reaction time, Go accuracy, No-Go accuracy, and an inhibitory control composite score (IC). The IC score was calculated as the product of mean Go accuracy and mean No-Go accuracy. Higher accuracy and IC values indicate better inhibitory control. Reaction time indices generated by the Early Years Toolbox were retained for both Go and No-Go trials. For Go trials, shorter reaction times indicate faster correct responding. For No-Go trials, the reaction time index incorporates the full 1,500-ms stimulus window on trials in which the response was correctly withheld; consequently, longer No-Go reaction times reflect a greater number of correctly withheld trials and therefore better inhibitory control. No-Go reaction time was interpreted together with No-Go accuracy and the inhibitory control composite score.

**Figure 2:**
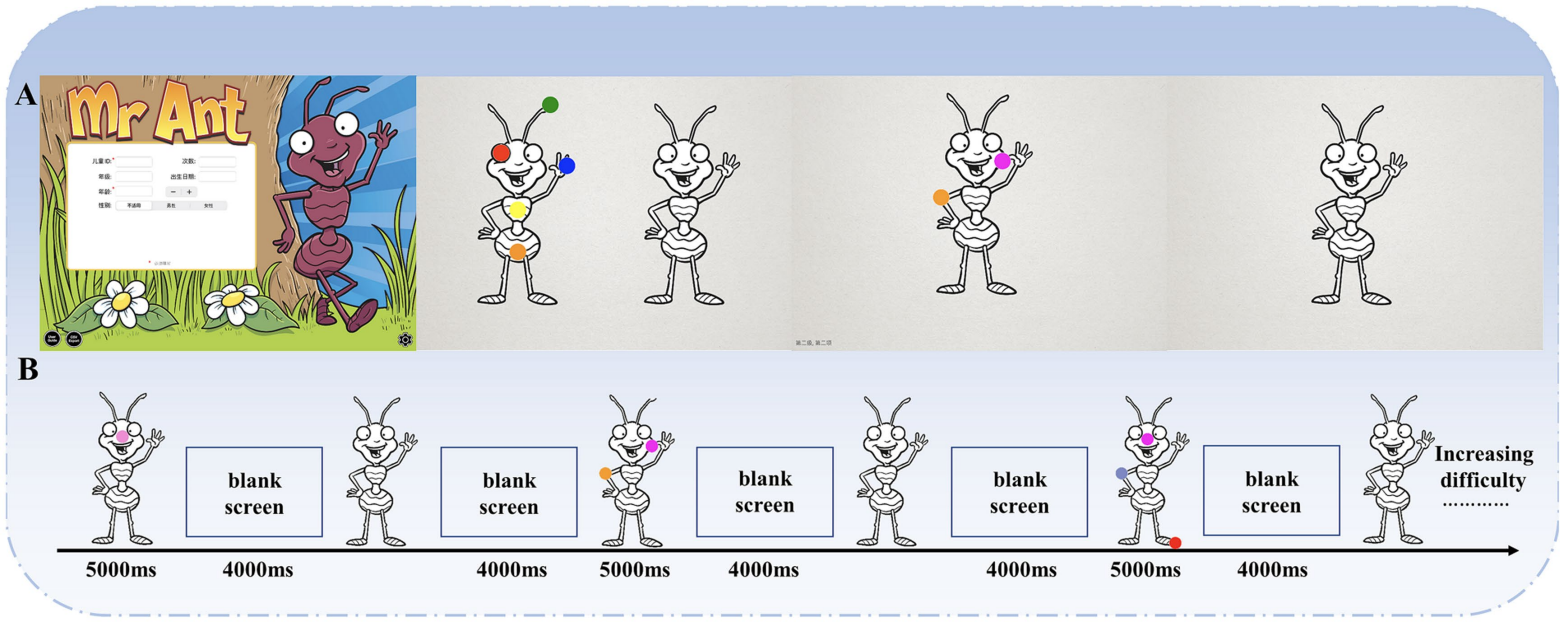
Example of the working memory task paradigm. **A** shows the paradigm instructions and examples of picture stimuli; **B** shows the flowchart of the formal test.

The Mr. Ant task was used to assess visuospatial working memory. Children viewed a cartoon character with colored stickers placed on different body locations for 5,000 ms, followed by a 4,000-ms blank screen. The character then reappeared without stickers, and children were asked to indicate the original sticker locations by tapping the screen. Task difficulty increased from one sticker to eight stickers, with three trials at each level (see Figure 2). If a child failed all three trials at the same level, the task was terminated. The total score was used to represent working-memory performance, with higher scores indicating better performance.

### 2.4 Exercise intervention protocol

The exercise intervention was administered individually (one child at a time) on a child-sized cycle ergometer suitable for children aged 3–12 years. The cycle ergometer was selected because it allows standardized aerobic exercise with precise control of workload and continuous heart-rate monitoring (Takken et al., 2017; Takken and Hulzebos, 2024).

Preparation phase: Before each session, the investigator ensured that the cycle ergometer was functioning properly and adjusted the seat height and angle so that each child could cycle comfortably. Children were screened for exercise contraindications and encouraged to wear suitable sports clothing and shoes (Riebe et al., 2015). The investigator briefly introduced the equipment and safety procedures to the child and guardian. A calisthenics warm-up was then completed, consisting of clapping in front of the chest × 8, overhead clapping × 8, forward arm circles × 8, backward arm circles × 8, squats × 8, and squat jumps × 8.

Exercise phase: The intervention consisted of continuous aerobic cycling within the target heart-rate range. After the warm-up, participants wore a Polar heart-rate monitor and cycled on the ergometer in the intervention room of the children’s hospital. The investigator adjusted resistance as needed to maintain the target heart-rate range for each condition. To support engagement and adherence, the investigator remained beside the child throughout each cycling session and provided standardized verbal encouragement. Before each session, children were told that they would receive small stickers and access to animated picture books after completing the exercise session. These rewards were provided after session completion. During cycling, heart rate and behavioral signs were monitored continuously. When heart rate exceeded the target range or when the child showed signs of fatigue or discomfort, the investigator temporarily reduced the ergometer workload or allowed a brief rest if needed, and then adjusted the workload again to return heart rate to the target range. No game-like or cognitively demanding stimuli were provided during cycling, because the purpose of the protocol was to isolate the effects of exercise intensity and duration. Exercise intensity was monitored continuously with the Polar heart-rate monitor and controlled using the heart rate reserve (HRR) method based on the Karvonen formula: target heart rate = resting heart rate + [(maximum heart rate − resting heart rate) × target intensity]. Maximum heart rate was estimated as 220 − age. Low intensity was defined as 40–50% HRR, whereas moderate intensity was defined as 60–70% HRR. (Garber et al., 2011; She et al., 2015; Yabe et al., 2021). These intensity ranges were used as operational definitions of low- and moderate-intensity exercise in the present study, considering the participants’ age, clinical characteristics, exercise tolerance, and safety during repeated acute exercise sessions. Throughout the session, the investigator closely monitored heart rate, breathing frequency, facial expression, and posture. If any sign of fatigue, discomfort, or abnormal reaction emerged, the exercise was stopped immediately and rest was provided.

Cool-down phase: Children gradually reduced cycling speed before stopping. Simple stretching and relaxation exercises involving the neck, shoulders, waist, legs, arms, and fingers were then completed to relieve fatigue. Mean exercise heart rate was recorded.

### 2.5 Statistical analyses

All statistical analyses were conducted in SPSS 26.0. Outcome variables were first examined for distributional assumptions. Mauchly’s test of sphericity was then used for repeated-measures outcomes (Mauchly, 1940). When the sphericity assumption was met, results are reported using the original degrees of freedom. When the assumption was violated, Greenhouse–Geisser-corrected degrees of freedom were used (Greenhouse and Geisser, 1959).

Repeated-measures ANOVAs were conducted to examine differences across the five experimental conditions. When a significant main effect of condition was observed, planned comparisons were conducted to compare each exercise condition with the sedentary control condition. For each outcome, the four planned comparisons were corrected using the Benjamini–Hochberg false discovery rate (FDR) procedure (Benjamini and Hochberg, 1995). FDR-adjusted *p* values are reported as *q* values. Statistical significance was set at *p* < 0.05 for omnibus ANOVAs and *q* < 0.05 for planned comparisons. Effect sizes are reported as *partial η^2^*, with 0.01, 0.06, and 0.14 representing small, medium, and large effects, respectively (Cohen, 2013; Lakens, 2013).

## 3 Results

### 3.1 Demographic data and exercise monitoring

Thirty participants were included in the final analyses, including 25 boys and 5 girls. The mean chronological age was 5.58 ± 1.82 years, and the mean CARS score was 33.13 ± 1.98 (ranged from 30 to 36), indicating a sample with generally mild-to-moderate ASD symptom severity. Mean heart rate increased in accordance with exercise intensity, with higher values observed in the moderate-intensity conditions than in the low-intensity conditions (see Table 1). All participants completed all four cycling sessions, including both 40-min cycling conditions. No session was terminated early, and no adverse events were observed. Mean heart rates in the low- and moderate-intensity conditions were consistent with the corresponding target intensity ranges, indicating that the prescribed exercise intensities were generally achieved during the cycling sessions.

**Table 1:** Demographic characteristics and heart-rate monitoring across conditions.

Variable	Sedentary control	20 min Low	40 min Low	20 min Moderate	40 min Moderate
*n*	30	30	30	30	30
Sex (boys/girls)	25/5	25/5	25/5	25/5	25/5
Age, years	5.58 ± 1.82	5.58 ± 1.82	5.58 ± 1.82	5.58 ± 1.82	5.58 ± 1.82
CARS score	33.13 ± 1.98	33.13 ± 1.98	33.13 ± 1.98	33.13 ± 1.98	33.13 ± 1.98
Heart rate, bpm	88.67 ± 5.39	150.15 ± 6.68	152.50 ± 5.81	171.96 ± 9.36	176.00 ± 8.89

Values are presented as mean ± SD. CARS, Childhood Autism Rating Scale; bpm, beats per minute.

### 3.2 Differences in executive function across conditions

Repeated-measures ANOVAs were conducted to examine the effects of intervention condition on executive function in children with ASD. The descriptive statistics of executive function scores of children with autism spectrum disorder after different intervention conditions are shown in Table 2.

**Table 2:** Executive-function performance across intervention conditions.

Variable	Sedentary control	20 min Low	40 min Low	20 min Moderate	40 min Moderate
Go RT, s	1.032 ± 0.150	1.028 ± 0.154	1.009 ± 0.177	1.036 ± 0.135	0.936 ± 0.133
No-Go RT, s	1.155 ± 0.259	1.149 ± 0.266	1.247 ± 0.191	1.280 ± 0.176	1.257 ± 0.213
Go ACC	0.739 ± 0.185	0.771 ± 0.163	0.778 ± 0.170	0.775 ± 0.214	0.848 ± 0.193
No-Go ACC	0.594 ± 0.272	0.642 ± 0.241	0.676 ± 0.200	0.737 ± 0.194	0.725 ± 0.235
IC score	0.440 ± 0.235	0.496 ± 0.209	0.534 ± 0.211	0.574 ± 0.231	0.621 ± 0.264
WM score	4.367 ± 0.928	4.367 ± 0.809	4.433 ± 0.858	4.533 ± 0.860	4.767 ± 1.040

Values are presented as mean ± SD. RT, reaction time; ACC, accuracy; IC, inhibitory control composite score; WM, working-memory score.

**Figure 3:**
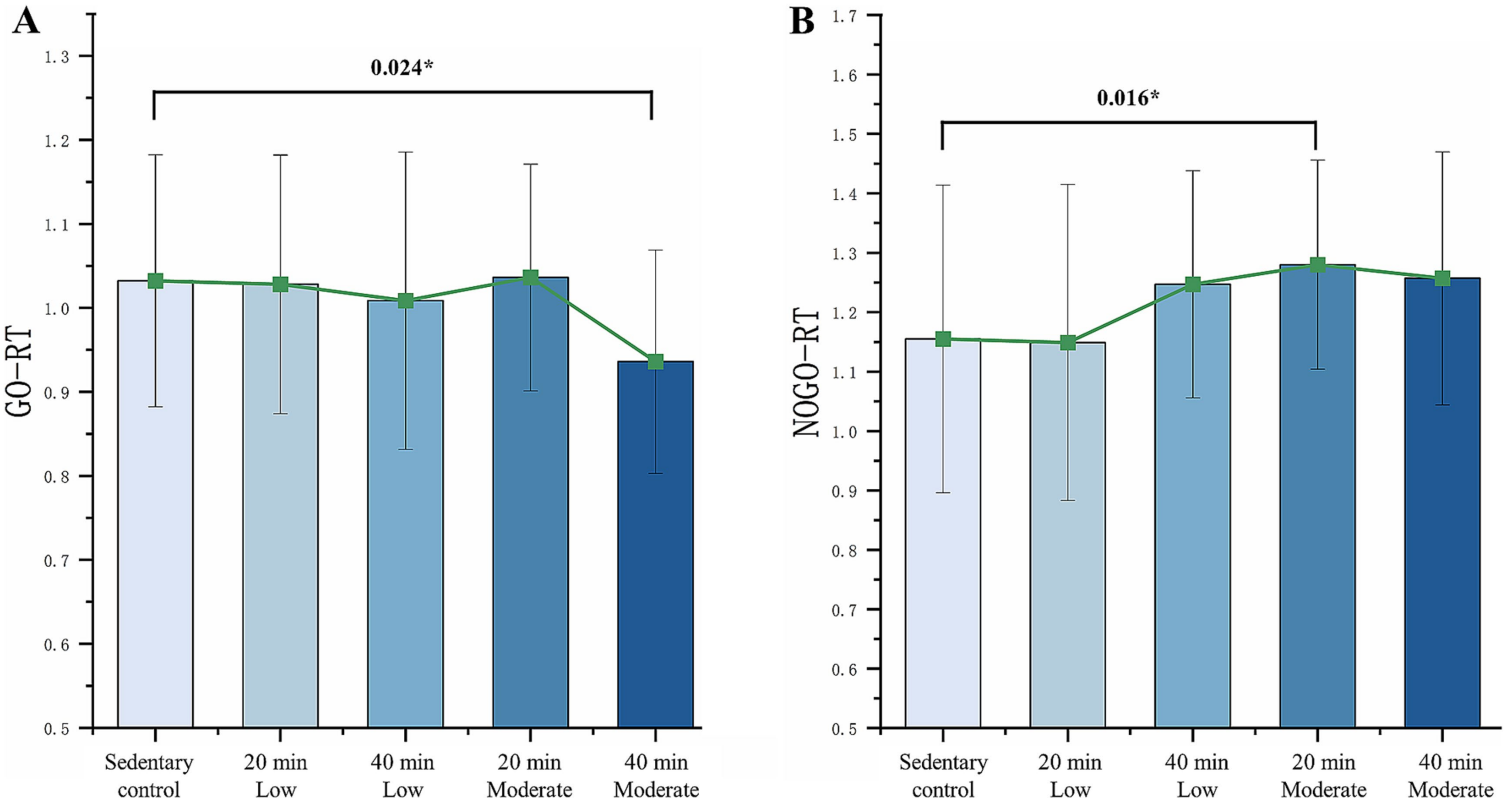
Reaction time performance on the inhibitory control task under different conditions. **A** shows reaction times under the Go condition; **B** shows reaction times under the No-Go condition; * indicate the level of significance.

For Go reaction time, Mauchly’s test indicated that the sphericity assumption was met (*W* = 0.767, *p* = 0.608). A significant main effect of condition was observed, *F*_(4, 116)_ = 2.921, *p* = 0.024, *partial η^2^* = 0.092. FDR-corrected planned comparisons showed that the 40-min moderate-intensity condition was significantly shorter than that in the sedentary control condition, *q* = 0.024 (see Figure 3A).

For No-Go reaction time, sphericity was also met (*W* = 0.608, *p* = 0.137). A significant main effect of condition was found, *F*_(4, 116)_ = 3.822, *p* = 0.006, *partial η^2^* = 0.116. FDR-corrected planned comparisons showed that 20-min moderate-intensity exercise was significantly longer than that in the sedentary control condition, *q* = 0.016. The comparison between 40-min moderate-intensity exercise and the sedentary control condition did not survive FDR correction, *q* = 0.080 (see Figure 3B).

**Figure 4:**
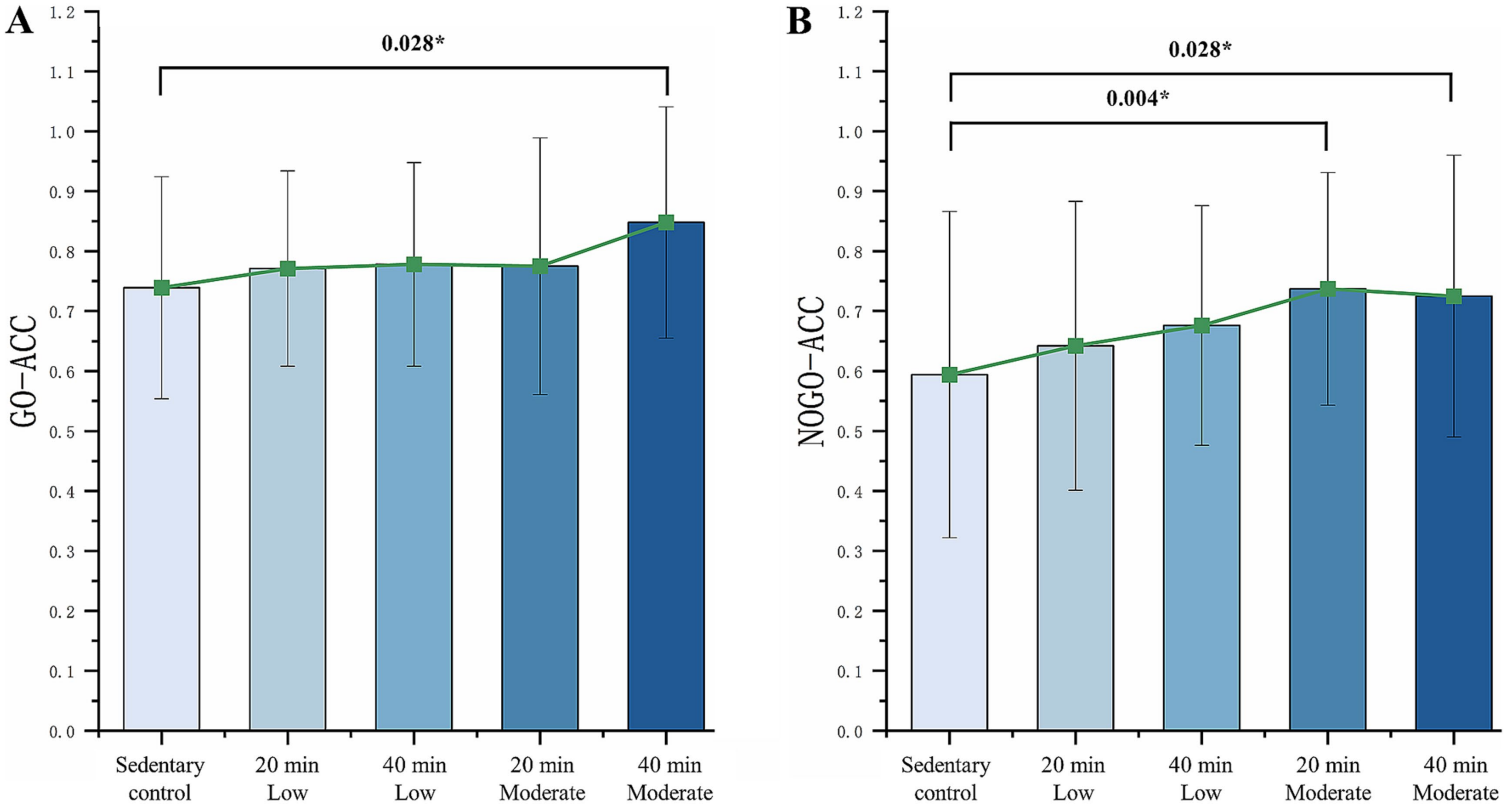
Accuracy performance on the inhibitory control task under different conditions. **A** shows accuracy under the Go condition; **B** shows accuracy under the No-Go condition; * Indicate the level of significance.

For Go accuracy, the sphericity assumption was met (*W* = 0.670, *p* = 0.278), and the main effect of condition was significant, *F*_(4, 116)_ = 2.507, *p* = 0.046, *partial η^2^* = 0.080. FDR-corrected planned comparisons indicated that the 40-min moderate-intensity condition was significantly higher than the sedentary control condition, *q* = 0.028 (see Figure 4A).

For No-Go accuracy, sphericity was met (*W* = 0.575, *p* = 0.087). The main effect of condition was significant, *F*_(4, 116)_ = 3.778, *p* = 0.006, *partial η^2^* = 0.115. FDR-corrected planned comparisons showed that both 20-min moderate-intensity exercise, *q* = 0.004, and 40-min moderate-intensity exercise, *q* = 0.028, were significantly higher than the sedentary control condition (see Figure 4B).

**Figure 5:**
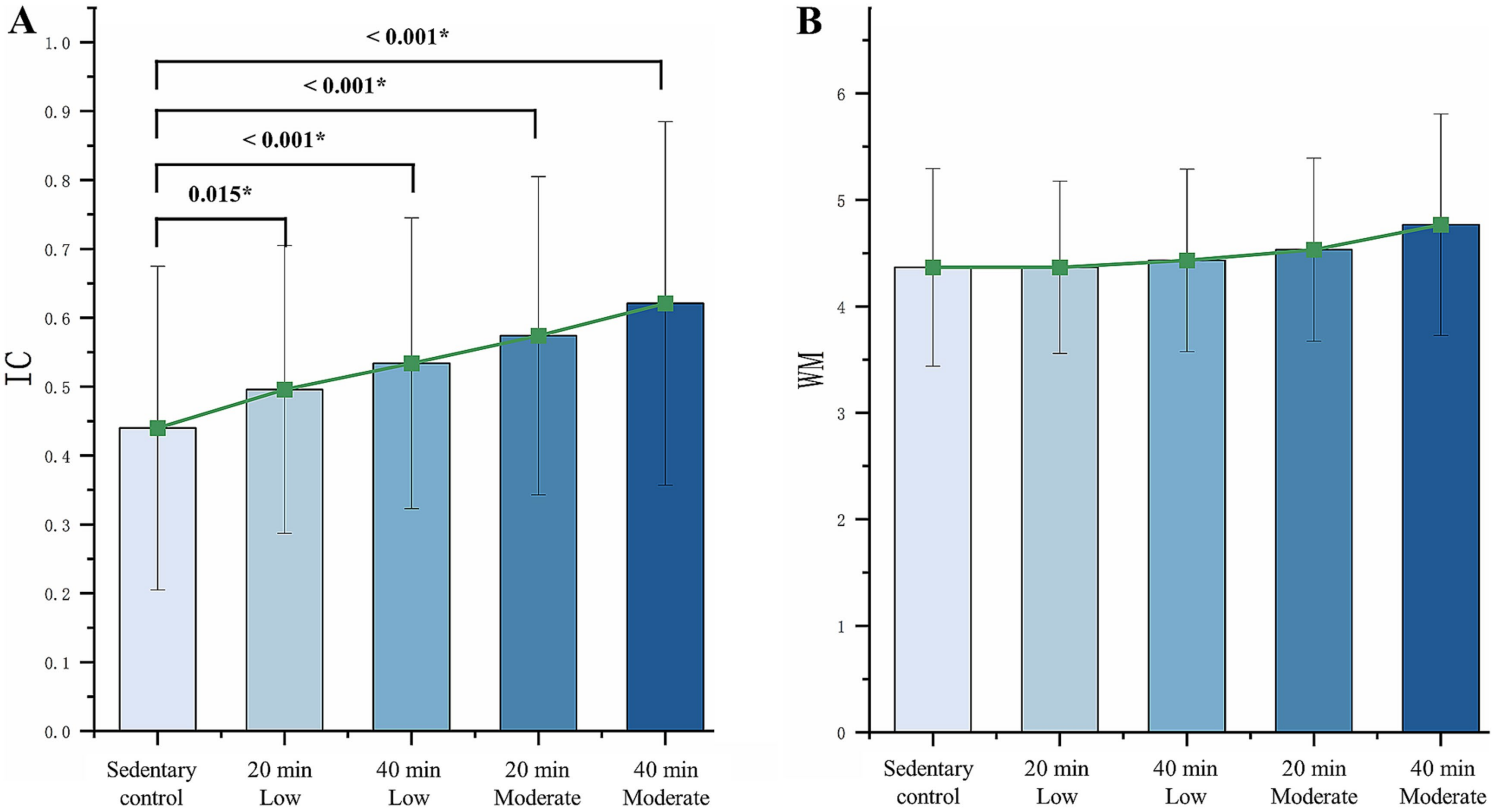
Inhibitory control scores and working memory scores under different conditions.**A** shows inhibitory control scores; **B** shows working memory scores; * indicate the level of significance.

For the inhibitory control composite score, the sphericity assumption was violated (*W* = 0.079, *p* < 0.001), so Greenhouse–Geisser-corrected results were used (*ε* = 0.505). The corrected ANOVA revealed a significant main effect of condition, *F*_(2.021, 58.612)_ = 19.771, *p* < 0.001, *partial η^2^* = 0.405. FDR-corrected planned comparisons showed that all four exercise conditions were significantly higher than the sedentary control condition, including 20-min low-intensity exercise, *q* = 0.015, 40-min low-intensity exercise, *q* < 0.001, 20-min moderate-intensity exercise, *q* < 0.001, and 40-min moderate-intensity exercise, *q* < 0.001 (see Figure 5A).

For working memory, the sphericity assumption was met (*W* = 0.675, *p* = 0.291), but the main effect of condition was not significant, *F*_(4, 116)_ = 1.114, *p* = 0.353, *partial η^2^* = 0.037. Therefore, planned comparisons were not interpreted for working memory (see Figure 5B).

## 4 Discussion

The present study used a within-subject repeated-measures design to examine whether acute cycling with different combinations of intensity and duration was associated with differential post-session executive-function performance in children with ASD. The main finding was that inhibitory control, but not working memory, differed significantly across conditions. Among the tested exercise doses, moderate-intensity cycling showed the clearest post-session advantages relative to sedentary control, and the 40-min moderate-intensity condition showed the most consistent pattern across inhibitory-control outcomes.

### 4.1 Effects on inhibitory control

The present findings indicate that inhibitory control in children with ASD is sensitive to acute exercise. Under Go conditions, 40 min of moderate-intensity exercise was significantly better than the sedentary control condition in both reaction time and accuracy. Under No-Go conditions, moderate-intensity exercise also showed advantages, with 20 min of moderate-intensity exercise producing longer No-Go reaction times, reflecting a greater number of correctly withheld trials, and both 20 min and 40 min of moderate-intensity exercise improving No-Go accuracy. In addition, the inhibitory control composite score showed the broadest facilitation pattern, with all four exercise conditions yielding higher scores than the sedentary control condition.

This pattern is broadly consistent with previous work showing that acute aerobic exercise can facilitate executive functioning, especially in tasks that require efficient response regulation (Bremer et al., 2020; Chang et al., 2012; Liu et al., 2020). Inhibitory control is closely related to attentional control and behavioral suppression, both of which may benefit from exercise-induced changes in arousal, neural activation, and resource allocation (Bari and Robbins, 2013; Diamond, 2013; Hillman et al., 2009; McMorris, 2016).

Several mechanisms may help explain why acute cycling was associated with better inhibitory-control performance. First, acute aerobic exercise can increase physiological arousal and catecholaminergic activity, which may enhance attentional readiness and response regulation during executive tasks (McMorris, 2016). This arousal-related explanation is consistent with the observation that moderate-intensity cycling showed clearer benefits than low-intensity cycling in several inhibitory-control outcomes. Second, acute exercise may increase cerebral blood flow and oxygen delivery, thereby providing a more favorable neural context for executive processing. Evidence from active and passive exercise studies suggests that post-exercise increases in cerebral blood flow may contribute to short-term executive-function benefits (Shirzad et al., 2022). Third, inhibitory control depends strongly on prefrontal and frontoparietal control networks, and acute exercise may transiently modulate cortical activation and excitability in these systems. For example, light aerobic exercise has been shown to modulate cortical excitability and selected executive-function tasks (Morris et al., 2020), while studies in children have linked acute exercise to changes in neural indices of cognitive control (Drollette et al., 2014; Hillman et al., 2009). In children with ASD, exploratory work has further shown that acute exercise can alter prefrontal oxygenation during inhibitory-control performance (Bremer et al., 2020). Therefore, the present findings may reflect, at least in part, exercise-induced changes in arousal regulation, cerebral perfusion, prefrontal activation, and executive-control network efficiency. However, because neurophysiological measures were not collected in the present study, these interpretations remain tentative.

From the perspective of intensity, the present results suggest that moderate-intensity cycling produced more consistent detectable inhibitory-control gains than low-intensity cycling under the present protocol. This finding accords with the optimal-intensity hypothesis, according to which moderate levels of physiological arousal may be most favorable for cognitive processing, whereas lower intensities may not be sufficient to trigger robust cognitive benefits (McMorris, 2016; Smith et al., 2016; Tsai et al., 2021). Importantly, this does not imply that low-intensity exercise is ineffective; rather, low-intensity cycling may provide a smaller or less consistent physiological stimulus under the present protocol. From the perspective of duration, 20 min of moderate-intensity exercise was sufficient to improve No-Go reaction time, whereas both 20 min and 40 min of moderate-intensity exercise improved No-Go accuracy. In contrast, only 40 min of moderate-intensity exercise significantly improved Go reaction time and Go accuracy. This pattern suggests that when exercise intensity is adequate, longer duration may strengthen the cognitive facilitation effect (Cai et al., 2025; Cantelon and Giles, 2021; McMorris, 2016). Forty minutes of exercise may provide a longer window for the accumulation of exercise-induced physiological changes relevant to cognition.

The stronger effects observed in No-Go trials are also meaningful. Because Go stimuli dominate the task, participants develop a prepotent response tendency. No-Go trials require suppression of this dominant response and therefore place heavier demands on inhibitory control. Acute exercise effects may be more likely to emerge in conditions with greater executive-control demands (Chang et al., 2012; Drollette et al., 2014; Hillman et al., 2009), which may explain why the No-Go metrics were particularly responsive.

### 4.2 Effects on working memory

In contrast to inhibitory control, working memory did not differ significantly across exercise conditions. This result suggests that the immediate effects of acute exercise on visuospatial working memory may be limited in young children with ASD, at least under the task conditions used here (Habib et al., 2019; Liang et al., 2022).

One possible explanation concerns task demands and developmental suitability. The Mr. Ant task requires children to encode, retain, and reproduce visuospatial locations after a delay, and task difficulty increases progressively across trials. For young children with ASD, especially those with a mean age of approximately 5–6 years, this task may place substantial demands on comprehension, sustained attention, and visuospatial maintenance. If the task was relatively difficult for some participants, performance may have been constrained by floor effects or limited score variability, thereby reducing sensitivity to detect subtle post-exercise differences (Habib et al., 2019; Howard and Melhuish, 2017; Šimkovic and Träuble, 2019; Terwee et al., 2007). A second explanation is that working memory may have a different temporal and neurocognitive response profile from inhibitory control. Inhibitory-control tasks often require rapid response selection and suppression of a prepotent response, processes that may be especially sensitive to acute changes in arousal, attentional readiness, and response regulation. By contrast, visuospatial working memory requires the maintenance and manipulation of information across a delay and may rely on more sustained frontoparietal coordination. Therefore, a single bout of cycling may be sufficient to influence response-regulation processes but not necessarily sufficient to produce an immediately observable behavioral improvement in a demanding working-memory task (Benzing et al., 2018; Cai et al., 2025; Cantelon and Giles, 2021; Liang et al., 2022).

The timing of post-exercise assessment may also have contributed to the null working-memory finding. Acute exercise effects on cognition can vary according to when the task is administered after exercise, and different EF components may show different time courses of facilitation or recovery. It is possible that the assessment window used in the present study was more sensitive to inhibitory-control changes than to working-memory changes. Future studies should consider multiple post-exercise testing windows to determine whether working-memory performance changes at later or more specific time points after exercise.

Finally, the absence of behavioral improvement does not rule out the possibility of underlying neurophysiological modulation. Acute exercise may alter cerebral blood flow, cortical activation, or neural efficiency without producing an immediate measurable change in task score, particularly when the behavioral task is demanding or when the sample is developmentally heterogeneous. Because the present study did not include neurophysiological measures, it remains unclear whether working-memory-related neural processes were unaffected or whether neural changes occurred without behavioral expression. Future studies combining behavioral tasks with measures such as fNIRS, EEG, or cerebral blood-flow indices may help clarify whether acute exercise influences working-memory networks in children with ASD even when overt performance remains stable (Bremer et al., 2020; Cai et al., 2025; Hillman et al., 2009; Morris et al., 2020).

## 5 Strengths and limitations

This study has several strengths. The within-subject design reduced the influence of stable individual differences, the Latin square counterbalancing minimized order effects across the four exercise sessions, and the 7-day interval between sessions helped reduce carryover effects. In addition, both exercise intensity and duration were manipulated simultaneously, allowing a more refined examination of dose-related effects than is typical in the existing literature.

Several limitations should also be acknowledged. First, the sedentary control session was completed at enrollment and was not counterbalanced with the four exercise sessions. This design feature may have introduced familiarization, learning, or order effects, because children may have become more accustomed to the testing environment, task procedures, and investigator instructions during later exercise sessions. Although the order of the four exercise sessions was counterbalanced using a Latin square design and sessions were separated by at least 7 days, these procedures only reduced order and carryover effects among the exercise conditions and did not eliminate the potential bias associated with administering the sedentary control session first. In addition, the sedentary control session was based on heart-rate stabilization rather than a fixed seated duration, and the exact time required to reach stabilization was not systematically recorded. Although this approach helped ensure a physiologically stable resting state before testing, variability in quiet sitting duration may have influenced boredom, fatigue, or attentional state. Therefore, comparisons between exercise conditions and the sedentary control condition should be interpreted cautiously, as they may partly reflect greater task familiarity in later sessions. This limitation may have led to an overestimation of the apparent advantage of exercise conditions relative to sedentary control. Future studies should use a fully counterbalanced crossover design with a fixed-duration sedentary control condition, or record time to heart-rate stabilization and include it as a potential covariate. Second, the sample size was modest, which may limit generalizability. Although ASD symptom severity was characterized using CARS and the sample was generally within the mild-to-moderate range, ADHD symptom severity was not assessed dimensionally using a standardized ADHD-specific scale. Therefore, the possible influence of subclinical attentional or hyperactivity symptoms on executive-function performance cannot be fully ruled out. Third, only inhibitory control and working memory were assessed; cognitive flexibility was not analyzed because of task-comprehension difficulties in this age group. Consequently, the findings should be interpreted as reflecting selected executive-function components rather than global executive function. Fourth, although the within-subject design strengthened internal control, the study was not a randomized controlled trial with independent groups. Fifth, the study focused on the immediate effects of acute exercise, so the long-term effects of repeated exposure to different exercise doses remain unclear. In addition, executive function was not assessed immediately before each exercise session. Therefore, day-to-day variation in baseline cognitive performance could not be fully controlled, even though sessions were scheduled at the same time of day and separated by at least 7 days. Sixth, no neurophysiological measures, such as prefrontal oxygenation, cortical activation, cerebral blood flow, or electrophysiological indices, were collected. Therefore, the proposed mechanisms involving arousal regulation, cerebral perfusion, cortical excitability, and executive-control networks remain speculative. Seventh, standardized subjective responses after each session were not assessed. Although heart rate, behavioral signs, session completion, and adverse events were monitored, we did not include child-reported perceived exertion, affective state, enjoyment, or happiness after each condition. Because reliable subjective ratings can be challenging in young children with ASD, future studies should use developmentally appropriate and ASD-sensitive tools, such as child-friendly RPE scales, visual affective scales, or observer-assisted reports. Finally, the feasibility and generalizability of the longer exercise dose should be interpreted cautiously. In the present supervised protocol, all participants completed both 40-min cycling conditions, mean heart rates remained within the target intensity ranges, and no adverse events were observed. Engagement was supported through continuous monitoring, verbal encouragement, individualized workload adjustment, and small rewards after session completion. These observations suggest that the 40-min cycling sessions were feasible under closely supervised clinical or laboratory conditions. Future studies should include structured adherence and engagement measures and examine whether shorter, more ecologically practical exercise bouts produce comparable cognitive benefits in school or community settings.

## 6 Conclusion

Acute cycling may have selective short-term effects on selected executive function components in children with ASD. Inhibitory control, but not working memory, differed significantly across exercise conditions. Moderate-intensity cycling showed the clearest post-session advantages relative to sedentary control, and the 40-min moderate-intensity condition showed the most consistent pattern across inhibitory-control outcomes. These findings highlight the importance of considering exercise dose when designing cognitive-oriented exercise activities for children with ASD, while also indicating that future fully counterbalanced crossover studies are needed to confirm the optimal acute-exercise prescription.

## Data Availability

The datasets presented in this article are not readily available because the anonymized datasets are available from the corresponding author upon reasonable request. Access is restricted because the data involve child participants with autism spectrum disorder and may contain potentially sensitive information; therefore, data sharing will be subject to ethical and institutional requirements. Requests to access the datasets should be directed to Dr. Yifan Shi, dx120220088@stu.yzu.edu.cn.
