# Supplementary material for: Dose-related effects of acute cycling on executive function in children with autism spectrum disorder: a within-subject repeated-measures study
Source: Front Psychol. 2026 Jul 30;17:1902127. doi: 10.3389/fpsyg.2026.1902127 (PMC13467928; doi:10.3389/fpsyg.2026.1902127)
Supplement: Supplementary file 1 [file Data_Sheet_1.DOCX]

# CONSERVE-CONSORT Checklist — Manuscript 1902127

**Study:** Dose-Related Effects of Acute Cycling on Executive Function in Children with Autism Spectrum Disorder: A Within-Subject Repeated-Measures Study

**Trial registration:** Chinese Clinical Trial Registry, ChiCTR2400091872 (registered 5 November 2024; first enrollment 1 March 2025; last assessment December 2025)

**Design:** Within-subject repeated-measures — one non-exercise sedentary control session plus four counterbalanced cycling conditions in a 2 (intensity) × 2 (duration) dose matrix. Every participant received every condition.

**Reference:** CONSERVE 2021 Statement (Orkin et al., JAMA. 2021;326(3):257–265).

*Page locations refer to the revised manuscript (manuscript_Revised3.pdf).*

**Part A — CONSERVE-specific sections**

| **#** | **CONSERVE section** | **Author response** |
| --- | --- | --- |
| **I** | Extenuating circumstances — describe the circumstances and how they constitute extenuating circumstances | None. The trial was designed and conducted as prospectively registered (ChiCTR2400091872). No extenuating circumstance (e.g., pandemic, natural disaster, civil unrest, regulatory suspension, or change in the standard of care) affected the design or conduct of the study. |
| **II** | Important modifications — impacts, mitigating strategies, rationale, and modification timeline | None. No modifications were made to the trial design, eligibility, interventions, outcomes, or analysis in response to any extenuating circumstance. The differences between the public registration record and this manuscript reflect partial reporting of Study 1 of a broader registered project, not a modification to the trial. |
| **III** | Responsible parties — who planned, reviewed, and approved modifications | Not applicable. No modifications were made in response to extenuating circumstances. |
| **IV** | Interim data — whether accumulating data informed modifications; whether examined by study group; whether reviewers were blinded to allocation | Not applicable. No accumulating outcome data were used to inform any modification. |

**Part B — Core CONSORT 2010 items and their status under extenuating circumstances**

*Status key: U = unaffected by extenuating circumstances; A = directly affected; M = modified as a mitigating strategy. All items below are U.*

| **No.** | **CONSORT 2010 item** | **Reported (section)** | **Status** |
| --- | --- | --- | --- |
| 1a | Identification as a randomised trial in the title | Title — described as a within-subject repeated-measures study (not an independent-group RCT) | **U** |
| 1b | Structured summary of trial design, methods, results, and conclusions | Abstract | **U** |
| 2a | Scientific background and rationale | Introduction | **U** |
| 2b | Specific objectives or hypotheses | Introduction (final paragraph) | **U** |
| 3a | Description of trial design including allocation ratio | Methods — Experimental Design and Procedure (within-subject 2×2 dose matrix + sedentary control; order counterbalanced by Latin square; all participants received all conditions) | **U** |
| 3b | Important changes to methods after trial commencement, with reasons | Not applicable — no changes after commencement | **U** |
| 4a | Eligibility criteria for participants | Methods — Participants (inclusion / exclusion criteria) | **U** |
| 4b | Settings and locations where data were collected | Methods — Participants / Exercise Intervention Protocol (Children’s Hospital Affiliated with Nanjing Medical University) | **U** |
| 5 | Interventions for each group with sufficient detail to allow replication | Methods — Exercise Intervention Protocol (HRR/Karvonen method, Polar HR monitoring, intensity and duration definitions) | **U** |
| 6a | Completely defined pre-specified primary and secondary outcome measures | Methods — Behavioral Assessments (inhibitory control via Go/No-Go; working memory via Mr. Ant; Early Years Toolbox). Cognitive flexibility (Card Sorting) prespecified as not administered for developmental reasons; all measured EF components reported (see Methods and Discussion) | **U** |
| 6b | Any changes to trial outcomes after commencement, with reasons | Not applicable — no changes to outcomes | **U** |
| 7a | How sample size was determined | Methods — Participants (G*Power 3.1; RM-ANOVA; α = 0.05; power = 0.80; ES = 0.25 → n = 21; 30 enrolled) | **U** |
| 7b | Interim analyses and stopping guidelines | Not applicable — no interim analyses or stopping rules | **U** |
| 8a | Method used to generate the random allocation sequence | Within-subject design: condition order counterbalanced using a Latin square design (Methods — Experimental Design and Procedure) | **U** |
| 8b | Type of randomisation; details of any restriction | Latin square counterbalancing of session order; no parallel-group randomisation | **U** |
| 9 | Allocation concealment mechanism | Not applicable — every participant received every condition (within-subject) | **U** |
| 10 | Who generated the allocation sequence, enrolled participants, and assigned them | Investigator-administered enrollment and Latin square ordering (Methods) | **U** |
| 11a | Blinding — who was blinded after assignment | Outcome tasks computer-administered and automatically scored (Early Years Toolbox); participants and investigators not blinded to condition (open within-subject design) | **U** |
| 11b | If relevant, description of the similarity of interventions | Conditions differed only in exercise intensity and duration by design (Methods) | **U** |
| 12a | Statistical methods used to compare outcomes | Methods — Statistical Analyses (RM-ANOVA; Mauchly’s test; Greenhouse–Geisser correction; planned comparisons; Benjamini–Hochberg FDR) | **U** |
| 12b | Methods for additional analyses | Methods — Statistical Analyses (planned comparisons vs sedentary control; FDR-adjusted q values) | **U** |
| 13a | Numbers of participants allocated, receiving intended treatment, and analysed | Results — Demographic Data and Exercise Monitoring (30 enrolled; all completed all conditions; all 30 analysed) | **U** |
| 13b | Losses and exclusions after allocation, with reasons | Results — none (no losses; no early termination) | **U** |
| 14a | Dates defining the periods of recruitment and follow-up | Methods — Ethics/Registration statement (first enrollment 1 March 2025; testing completed December 2025) | **U** |
| 14b | Why the trial ended or was stopped | Not applicable — trial completed as planned | **U** |
| 15 | Baseline demographic and clinical characteristics | Results — Demographic Data (age, sex, CARS; Table 1) | **U** |
| 16 | Numbers analysed in each condition | Results — 30 participants across all five conditions (within-subject) | **U** |
| 17a | Outcomes and estimation (effect size and precision) | Results — Differences in Executive Function Across Conditions (F, p, partial η²; q values; Figures 3–5) | **U** |
| 17b | For binary outcomes, both absolute and relative effect sizes | Not applicable — continuous outcomes only | **U** |
| 18 | Results of any other analyses performed | Results — inhibitory-control composite score; working memory | **U** |
| 19 | All important harms or unintended effects | Results — "No session was terminated early, and no adverse events were observed." | **U** |
| 20 | Trial limitations, sources of potential bias | Discussion — Strengths and Limitations | **U** |
| 21 | Generalisability (external validity) | Discussion — Strengths and Limitations / interpretation | **U** |
| 22 | Interpretation consistent with results | Discussion / Conclusions | **U** |
| 23 | Registration number and name of trial registry | Clinical Trial Registration and Ethics statements (ChiCTR2400091872) | **U** |
| 24 | Where the full trial protocol can be accessed | Not stated in manuscript; full protocol available from the corresponding author on request | **U** |
| 25 | Sources of funding and other support | Funding (National Social Science Foundation of China, 23ATY008) | **U** |

*Summary: All core CONSORT items are unaffected by extenuating circumstances. No extenuating circumstance and no important modification occurred during the trial. The CONSERVE-specific sections are therefore completed as "none / not applicable." The differences between the public registration record and this manuscript reflect partial reporting of Study 1 of a broader registered doctoral project.*
